# Perception of uncertainty concerning illness and coping styles among parents of children with central nervous system tumors: a correlation analysis

**DOI:** 10.3389/fped.2025.1425001

**Published:** 2025-02-12

**Authors:** Bai Lan, Zhao Qinqin, Li Ying, Zhou Zhihuan

**Affiliations:** Department of Neurosurgery, State Key Laboratory of Oncology in South China, Guangdong Provincial Clinical Research Center for Cancer, Sun Yat-sen University Cancer Center, Guangzhou, China

**Keywords:** intracranial tumors, caregiver, children, parents, coping style, perception uncertainty in illness

## Abstract

**Purpose:**

To investigate the perception of uncertainty concerning illness among parents of children with central nervous system tumors and their coping styles and to analyze correlations to provide a basis for developing targeted intervention measures.

**Methods:**

We studied 108 parents of children with central nervous system tumors admitted from January 2023 to January 2024. Information about these children and their parents were analyzed using the modified Parents' Perception of Uncertainty in Illness Scale (PPUS) and the Coping Health Inventory for Parents to calculate correlations of parents' perceptions of uncertainty concerning illness and coping styles.

**Results:**

The total score of PPUS was 45.28 ± 8.428 for the parents of children. The most common coping styles were the maintenance of family unity, cooperation, and optimism. The correlation analysis revealed that parents' perception of uncertainty concerning illness negative correlated with coping style (*p* *<* 0.05).

**Conclusion:**

The parents of children with central nervous system tumors show substantial perception of uncertainty concerning the illness and are susceptible to lack of communication and expression. Positive, effective coping methods should be adopted to reduce uncertainty concerning illness to reduce the psychological pressure on parents.

## Highlights

• Central nervous system tumors are the most common solid tumors in children. In this paper, we report an status of the perception of uncertainty concerning illness among parents of children with central nervous system tumors and their coping styles.
• Further, we show that correlations to provide a basis for developing targeted intervention.
• This study can provide reference for clinical psychological nursing and Effective clinical psychological nursing makes the families of children better integrate into the society. Promoting children's families to actively cope with the disease can reduce family pressure and social burden.

## Introduction

Central nervous system (CNS) tumors, a group of tumors that originate from the tissues or structures within the central nervous system, are the most common type of solid tumors in children (1). In China, the incidence of pediatric CNS tumors is 21∼25.9/million, accounting for approximately 25% of all pediatric solid tumors (2), and these tumors are the most common cause of death among children with malignant tumors (3).

Parents, as the primary caregivers for children, face significant stress when dealing with the cognitive, emotional, and physical impairments caused by central nervous system (CNS) tumors in their children (4). The lack of sufficient knowledge about the disease, the unpredictability of treatment outcomes, and concerns about disease recurrence can lead to a strong sense of uncertainty about the disease.Perception of uncertainty concerning illness is a cognitive state characterized by a lack of ability to judge concepts related to the disease (5). It can be experienced as uncertainty when the decision-maker cannot assign values to objects and events or cannot make accurate predictions of the results (6). This variable hinders the definitive and comprehensive evaluation of events.If parents are troubled by the uncertainty of the disease, it will not only increase their own psychological burden, but also may prevent them from providing appropriate assistance to their children, thereby affecting the quality of care for the child (5).

Scholars both domestically and internationally have conducted extensive research on the sense of disease uncertainty (7), finding that it is associated with the caregiving burden, anxiety, depression, and other psychological factors of caregivers (8). Studies also show that coping strategies and social support can reduce the disease uncertainty among caregivers of patients with chronic diseases. Due to the particularity of CNS tumors, exploring the current status of disease uncertainty among parents of these children and its correlation with disease coping strategies may guide clinical workers to help parents better cope with the child's rapid disease changes and poor prognosis, providing better family and social support for the child's recovery.

## Materials and methods

### Objectives

To investigate the perception of uncertainty concerning illness among parents of children with central nervous system tumors and their coping styles and to analyze correlations to provide a basis for developing targeted intervention measures.

### Study design and setting

A cross-sectional study design was employed. The study was conducted in two inpatient departments at Sun Yat-sen University Cancer Center. Ethical approval was obtained from the Institutional Review Board of Sun Yat-sen University Cancer Center (B2024-133-01).

### Participants

Participants were recruited through convenience sampling.We included 108 parents of children with intracranial tumors admitted to our department, from January 2023 to January 2024, which was approved by the local hospital. Inclusion criteria were (1) children's diagnosis following the NCCN Guidelines for Central Nervous System Tumors; (2) ability to read and write, with no abnormalities of cognitive or mental status; and (3) voluntarily participated in this study. Exclusion criteria encompassed the parents who can't participate, such as dysfunctions in reading, writing, or cognition.

### Data collection

All study participants were recruited from tertiary grade-A hospitals in Guang Zhou. The researchers explained the purpose, significance, and contents of the research to parents, selected participants in accordance with the selection criteria, and administered an anonymous paper questionnaire to the participants. The questionnaire was prepared in accordance with standard guidelines and promised confidentiality of all research data. After collecting the questionnaires, invalid questionnaires were discarded. The elimination criteria were as follows: (a) incomplete questionnaires, (b) questionnaires showing apparent patterns in the responses, or (c) questionnaires in which all answers were the same. Among the 108 questionnaires collected, all 108 were valid, yielding an effective response rate of 100%.

### Sociodemographic questionnaire

The basic information questionnaire was developed by the researchers. This questionnaire was developed in alignment with this study's objectives and was validated using the content-validity method by the faculty members of the nursing school at Sun Yat-sen University.

It included two parts: (i) basic information of parents of children with central nervous system tumors (e.g., the relationship with the children, age, occupation, marital status, only child or not, residence, degree of education, family monthly income, medical expenses payment method, and religious beliefs); (ii) basic information of children (gender, age, and treatment time).

### Parents' perception of uncertainty in illness scale (PPUS) questionnaire

This questionnaire was compiled by Mishel in 1983 (9). It consists of four dimensions: perception of uncertainty in illness, uncertainty in information, lack of information, and unpredictability, for a total of 31 items. Higher scores indicate a higher perception of uncertainty in illness. The revised Chinese version was translated by Ye Zengjie of Guangzhou University of Traditional Chinese Medicine, and the dimensions were changed from 4 to 2, and tested for reliability and validity (10). The two dimensions are Vague and Lack of communication. The specificity and sensitivity of the scale were 0.773 and 0.708. Ye Zengjie permitted us to use the Chinese version.

### Coping health inventory for parents (CHIP) questionnaire

This questionnaire was compiled by McCubbin (11). It consists of three subscales (maintaining family solidarity, cooperation, and optimism; seeking support from society to maintain self-esteem and psychological stability; and understanding the disease through consulting medical staff and communicating with other parents), with a total of 45 items. Higher scores indicate more positive and effective coping styles. The Chinese version of CHIP was introduced into China by Li and Wei (12). The overall Cronbach coefficient of the Chinese version of CHIP was 0.91, and those of each subscale were 0.92, 0.80, and 0.70, respectively.

### Required sample size

The most important indicator in this study is the correlation coefficient between Parents' Perception of Uncertainty in Illness Scale (PPUS) and Coping health inventory for parents (CHIP) questionnaire. Sample size was estimated using G*Power version 3.1 software (Heinrich-Heine-Universität Düsseldorf, Düsseldorf, Germany). The expected correlation coefficient (r) is at least 0.3, with a type I error (alpha) of 0.05 and a power of 0.80, two-tailed test. The calculation results indicate that a minimum of 100 participants is required.

### Statistics

SPSS 20.0 statistical software was used for analysis. Measurement data were expressed as $\bar{x}\pm s$. Descriptive analysis included constituent ratio. Spearman correlation analysis was used to analyze the correlations between parents' uncertainties and coping styles. *p* < 0.05 or *p* < 0.01 meant that a difference was statistically significant.

## Results

A total of 108 complete and valid questionnaires were collected, without any incomplete or invalid questionnaires. As shown in Table 1, parents' ages ranged from 25 to 51 years old (mean = 34.00, SD = 6.05). Among them, there are 43 fathers and 65 mothers, with 81.5% of the children having siblings. In terms of residence, 12% of families reside in provincial capitals, while 57% live in urban areas and towns, and 35.2% reside in rural regions. Regarding parental education levels, the majority (69.5%) have attained education ranging from junior middle school to specialized secondary school. As for family finances and medical expenses coverage, over 90% of households possess medical insurance for reimbursement.

**Table 1:** Parents’ sociodemographic characteristics (*n* = 108), in January 2023-January 2024 in China.

Variable	Number (%)
Age (yrs)	
Gender	
Male	43 (39.8)
Female	65 (60.2)
Marital status	
Married	105 (97.2)
Divorced	2 (1.9)
Widowed	1 (0.9)
Only child or not	
Yes	20 (18.5)
No	88 (81.5)
Place of residence	
Provincial capital city	13 (12.0)
Other towns	57 (52.8)
Village	38 (35.2)
Degree of education	
Primary school	7 (6.5)
Junior middle school	46 (42.6)
High school/technical secondary school	29 (26.9)
Junior college	12 (11.1)
Bachelor degree or above	14 (13.0)
Monthly household income	
<3,000	18 (16.7)
3,000–5,000	32 (29.6)
5,000–10,000	36 (33.3)
>10,000	22 (20.4)
Payment method for medical expenses	
Self-funded	6 (5.6)
New rural cooperative medical System	63 (58.3)
Urban medical insurance	37 (34.3)
Commercial medical insurance	2(1.9)

### PPUS scores of parents of children with central nervous system tumors

As shown in Table 2, the total score of PPUS for parents of children with intracranial tumors was 45.28 ± 8.428 points.

**Table 2:** The score of each dimension and total score of uncertainty in parents of children with central nervous system tumors.

Items	Rating range	Score range	Scores ($\bar{x}\pm s$)
Vague	0–25	10–24	17.69 ± 3.27
Lack of communication	0–45	11–42	27.68 ± 5.93
Total score of uncertainty feeling	0–70	23–65	45.28 ± 8.43

### CHIP scale score of parents of children with central nervous system tumors

Table 3 shows the mean value of the frequency scale and role scale of the three CHIP subscales of parents. The mean values of frequency scale and role scale of CHIP subscales in parents ranked from high to low as follows: maintaining family solidarity, cooperation, and optimism; understanding the disease through consulting medical staff and communicating with other parents; and seeking support from society to maintain self-esteem and psychological stability. Maintaining family solidarity, cooperation and optimism was the primary coping style adopted by the parents of children with central nervous system tumors, usually associated with the belief that this was the most effective way. Table 4 lists the top eight coping styles among parents of children with central nervous system tumors. Parents believed that the coping style of “family members working together as a whole” would be more effective.

**Table 3:** Frequency scale and effect scale scores of CHIP subscale score of parents of children with central nervous system tumors.

Items	Score range (points)	Frequency of use	Score range (points)	Degree of action
Maintaining family solidarity, cooperation, and optimism	1–5	4.25 ± 0.94	0–3	2.45 ± 0.73
Seeking support from society to maintain self-esteem and psychological stability	1–5	3.11 ± 1.21	0–3	1.95 ± 0.96
Understanding the disease through consulting medical staff and communicating with other parents	1–5	3.60 ± 1.19	0–3	2.19 ± 0.86

**Table 4:** Top eight coping styles in parents of children with central nervous system tumors.

Items	Sequencing of the role	Degree of the role	Frequency sequencing	Frequency of use
Family members work together as a whole	1	2.71 ± 0.52	2	4.64 ± 0.60
Try to keep the family stable	2	2.67 ± 0.55	5	4.54 ± 0.72
Trust my partner to support myself and your children	3	2.67 ± 0.59	4	4.59 ± 0.71
Believe that my children can get the best treatment	4	2.66 ± 0.49	3	4.59 ± 0.63
Take children to the hospital regularly for examination	5	2.65 ± 0.64	8	4.32 ± 0.94
Put more effect into taking care of my children	6	2.64 ± 0.55	1	4.78 ± 0.54
Encourage sick children to be more independent	7	2.57 ± 0.69	7	2.57 ± 0.69
Believe that the hospital will put patients’ rights and interests in the first place	8	2.52 ± 0.63	6	4.43 ± 0.79

### Correlation analysis between perception uncertainty in illness and coping style in parents of children with central nervous system tumors

Spearman correlation analysis showed a negative correlation correlation between perception of uncertainty in illness and coping style among parents of children with central nervous system tumors (*p* < 0.05 or *p* *<* 0.01) (see as Table 5).

**Table 5:** Correlation between the perception of uncertainty in illness and coping style among parents of children with central nervous system tumors (r-values).

Items	Vague	Lack of communication	Total score of uncertainty
Maintaining family solidarity, cooperation, and optimism (subscale 1)			
Application frequency score	−0.187	−0.084	−0.165
Score of the role	−0.307**	−0.173	−0.284**
Seeking support from society to maintain self-esteem and psychological stability (subscale 2)			
Application frequency score	−0.314**	−0.214*	−0.305**
Score of the role	−0.344**	−0.160	−0.305**
Understanding the disease through consulting medical staff and communicating with other parents (subscale 3)			
Application frequency score	−0.112	−0.103	−0.119
Score of the role	−0.154	−0.093	−0.145
Total score of the role of disease coping style	−0.342**	−0.176	−0.310**
Total score of coping frequency of disease coping style	−0.273**	−0.177	−0.261**

* *P* < 0.05.

** *P* < 0.01.

## Discussion

The perception of uncertainty concerning illness among parents of children with central nervous system tumors is produced primarily from fuzzy dimensions. Childrens' health significantly predicts parental perception of uncertainty concerning the illness (13). Physicians generally provide diagnoses, treatment plans, and disease information during hospitalization. Timely communication helps reduce the perception of uncertainty to some extent. Because of the complexity of central nervous system tumors and recurrent symptoms in children, parents cannot accurately judge or establish causal relationships regarding their children's conditions during prolonged stays in the hospital. When the absence of markers characterizes these situations, parents prefer to regard each event as unpredictable and out of control. Several events and corresponding stimuli can induce this uncertainty; however, these events vary with the type of therapeutic schedules provided to their children (9). Diagnosis and treatment of central nervous system tumors are typically beyond parental understanding and prediction, which restricts parents' predictions of the future and aggravates the perception of uncertainty. Items with higher scores of perception of uncertainty and parental need for information suggest that medical staff must be aware of the perception of uncertainty concerning illness on the part of parents. Reducing the parental perception of uncertainty may require the transmission of information about the disease (e.g., drug therapy, complications, and prognosis).

Our results suggest that parents of children with central nervous system tumors usually adopt positive coping styles and are willing to devote energy to take care of their children and work together. However, parents are less likely to seek support from society and are not concerned with their psychological state. Traditionally, most Chinese parents are unwilling to trouble others (14); they are accustomed to concealing and suppressing negative emotions. By contrast, they are more willing to adopt psychological states such as persistence and belief, including that diseases can be overcome through firm belief. Furthermore, these parents usually do not communicate with other parents who have sick children, obtain information about the disease, or seek help.

Children are quite sensitive to the emotions of adults, especially their parents. When parents can cope with their feelings, they can better stabilize their children, which has a positive effect on disease recovery (15). In the present study, we found that parents most often adopted the coping style of maintaining family unity, cooperation, and optimism and believed that this style helped themselves and their families. This finding suggests a need for medical staff to guide parents to communicate information about the disease. This intervention, combined with increased communication with other parents, can improve disease awareness. It is also essential for parents to understand that seeking support from society can improve information acquisition and reduce negative emotions and burdens when caring for children.

We found a significant negative correlation between the perception of uncertainty in illness and the coping style of parents of children with intracranial tumors.The results are similar to previous studies (16). This showed that the higher the level of parental uncertainty, the less effective coping style, and the less effective coping style, the higher the level of parental uncertainty. In future clinical intervention,we can regularly carry out medical knowledge popularization and parent seminars to improve parents’ understanding of disease-related diagnosis and treatment and health care knowledge, reduce the uncertainty level of parents of children with children, This study suggests that medical staff can adopt appropriate interventions [such as mindfulness therapy (17), etc.] to encourage parents to seek external support and comfort, so as to maintain a positive attitude towards their children's disease situation and reduce the disease uncertain.

Childhood cancer is a chronic, life-threatening disease. Parents of children with central nervous system tumors have a higher perception of uncertainty concerning illness than those with leukemia, sarcoma, or lymphoma (16). Like the concept of stress, coping is an umbrella term that covers a broad set of variables (18, 19). “Internal health” is widely supported by modern nursing theory. It may be helpful for nurses to reveal the internal health power of nursing objects and make use of all available resources to avoid patients suffering from pain and injury and help them fight against pain (20). In general, there is a need for medical staff to improve daily physician-patient communication, to strengthen the practice of parental seeking of support from society, to explain disease status patiently, and to promote better physician-patient cooperation. Meeting these goals will improve the management of children with intracranial tumors.

### Limitations

This study had some limitations. First, most participants were from China, potentially limiting the applicability of the findings in different cultural contexts. The study was conducted in a single center, which may affect the generalizability of the results.Thus, future studies should include multi-regional, multi-center clinical investigations to increase the diversity of the sample. Second, the study period was relatively short (January 2023 to January 2024), and this study was a cross-sectional survey that only looked at the characteristics of parental disease uncertainty in children with central nervous system tumors, and a longitudinal study design can be used in the future to evaluate how illness uncertainty and coping strategies evolve over time.Additionally, self-reported data from parents may be subject to bias, which need us do more to control this bias in the future.

## Conclusion

This study showed that parents' disease uncertainty was negatively correlated with coping styles, indicating that the higher the level of parents' disease uncertainty, the more apt they were to adopt ineffective coping styles to face the disease. In order to ensure the quality of care for children and improve the effective coping style of parents, this paper provides more convincing reasons for medical staff to consider the psychological state and coping style of parents of children. For parents who have a strong sense of uncertainty about the disease, medical professionals should give information support and emotional support.

## Data Availability

The original contributions presented in the study are included in the article/Supplementary Material, further inquiries can be directed to the corresponding author.
